# PCR-Based Analytical Methods for Quantification and Quality Control of Recombinant Adeno-Associated Viral Vector Preparations

**DOI:** 10.3390/ph15010023

**Published:** 2021-12-24

**Authors:** Anna A. Shmidt, Tatiana V. Egorova

**Affiliations:** 1 Laboratory of Modeling and Gene Therapy of Hereditary Diseases, Institute of Gene Biology, Russian Academy of Sciences, 119334 Moscow, Russia; a.shmidt.marlin@gmail.com; 2 Marlin Biotech LLC, 354340 Sochi, Russia; 3 Center for Precision Genome Editing and Genetic Technologies for Biomedicine, Institute of Gene Biology, Russian Academy of Sciences, 119334 Moscow, Russia

**Keywords:** gene therapy, rAAV, quality control, qPCR, reference standard material, calibration standard, ddPCR

## Abstract

Recombinant adeno-associated viral vectors (rAAV) represent a gene therapy tool of ever-increasing importance. Their utilization as a delivery vehicle for gene replacement, silencing and editing, among other purposes, demonstrate considerable versatility. Emerging vector utilization in various experimental, preclinical and clinical applications establishes the necessity of producing and characterizing a wide variety of rAAV preparations. Critically important characteristics concerning quality control are rAAV titer quantification and the detection of impurities. Differences in rAAV constructs necessitate the development of highly standardized quantification assays to make direct comparisons of different preparations in terms of assembly or purification efficiency, as well as experimental or therapeutic dosages. The development of universal methods for impurities quantification is rather complicated, since variable production platforms are utilized for rAAV assembly. However, general agreements also should be achieved to address this issue. The majority of methods for rAAV quantification and quality control are based on PCR techniques. Despite the progress made, increasing evidence concerning high variability in titration assays indicates poor standardization of the methods undertaken to date. This review summarizes successes in the field of rAAV quality control and emphasizes ongoing challenges in PCR applications for rAAV characterization. General considerations regarding possible solutions are also provided.

## 1. Introduction

Recombinant adeno-associated virus (rAAV) is an increasingly important gene therapy vector. These vectors became popular due to their natural characteristics. First of all, wild-type AAVs are not associated with any human disease. At the same time, naturally occurring AAV serotypes are able to transduce different tissue and cell types in vitro and in vivo, and their categorization has expanded with creation of engineered capsids. AAVs’ replication deficiency without a helper virus, as well as their low immunogenicity, prolonged transgene persistence and low integration efficiency into known and safe location makes them even more attractive delivery vehicles [1,2].

To date, three AAV-based therapies have been approved for medical application: Glybera (alipogene tiparvovec, discontinued), Luxturna (voretigene neparvovec) and Zolgensma (onasemnogene abeparvovec). These products utilize rAAV serotypes 1, 2 and 9 for the delivery of a functional gene copy. Gene delivery by viral vector can either compensate for a malfunctioning gene (gene replacement therapy) or provide a new function to help fight a disease (gene addition therapy). Collectively, these gene therapy approaches are being tested in more than 200 clinical trials [3]. Another adeno-associated vector application field refers to designing of AAV-based vaccine preparations. Ability to induce long-lasting and strong humoral and cellular immune responses and safety of administration was previously shown for AAV vaccines directed against various viral pathogens in animal studies [4,5,6] and early clinical studies [7,8]. Meanwhile, researchers have also gained the ability to introduce constructs that can directly modify mutation sites or alter gene expression via rAAV delivery. These include recent advances in RNA interference (RNAi)-based gene silencing, splicing modulations, CRISPR/Cas9-based genome or base editing, as well as introducing epigenetic modifications. Proof of concept of these gene therapy techniques has demonstrated high versatility regarding rAAV as a delivery vehicle for experimental or therapeutic applications [9,10].

Numerous clinical trials of rAAV-based therapeutics, vaccine development and laboratory applications of vectors, require the production of diverse rAAV preparations in large quantities. Differences can rely on either capsid proteins or internal effector and regulatory sequences. Scientists generate engineered capsids aimed to deliver genetic constructs to distinct tissue and cells, increase vector circulation time and reduce the titer necessary for infection. Gene therapy strategies encompass more diseases and require the delivery of new genes and regulatory nucleic acids. Continuous modifications of existing expression cassettes and the introduction of new regulatory elements aim to control expression inside the target tissue, along with minimizing undesirable adventitious effects. Variability in resulting capsids and genetic constructs raises the question of standardized methods regarding rAAV analytics, primarily quantification. Titer measurement methods encountering viral particle (vp) and viral genome (vg, gc) number should be distinguished from methods estimating infectious titer. The first group combines ELISA, dot blotting and others to detect capsids, while the second uses PCR-based methods and nucleic acid staining without amplification. Cellular methods are utilized to estimate the number of infectious units. Wide characterization of rAAV preparations provide information about quantity, identity, stability, functionality and presence of substances that may potentially interfere with successful vector administration [11]. The majority of these parameters can also be qualified using PCR, which is widely used in laboratories all around the world. PCR methods are also very useful for vector biodistribution and shedding studies, while other approaches have also demonstrated high sensitivity [12,13].

The growing popularity of rAAV vectors, not only as gene therapy vehicles but as experimental tools, compels researchers to examine multiple methods for their quantitative and qualitative analyses. In this review, we focus on PCR-based methods for rAAV quality control (QC). The final product characteristics mainly depend on AAV nature and rAAV production method; thus, the next two sections are devoted to these issues. Section 4 is dedicated to quantification assays, providing information about vector titer as one of the most important analytical criteria. In Section 5, we address the utilization of PCR methods for the quantification of possible contaminants of rAAV samples, relying on the detection of DNA impurities. Recommendations for the sufficient characterization of rAAV preparations based on a complex approach and future perspectives are provided in Section 6 and Section 7.

## 2. AAV Biology

**Figure 1:**
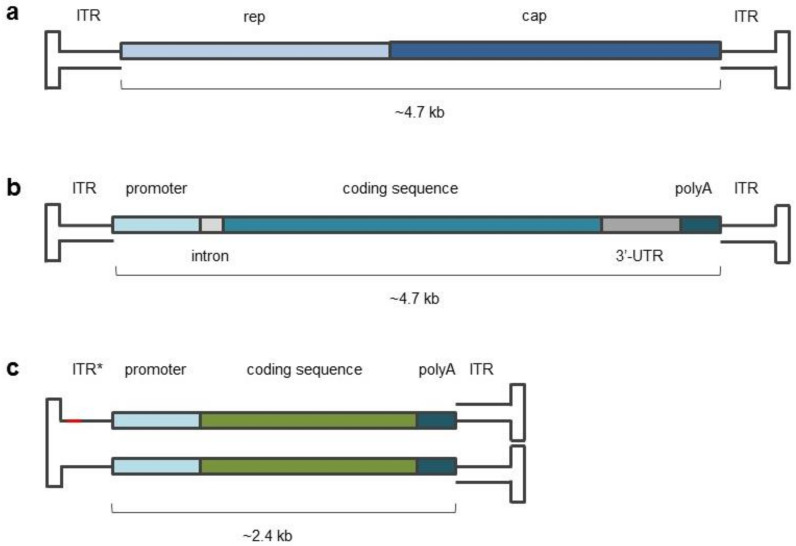
Structure of wild-type and vector AAV genomes. (**a**) Map of the wild-type genome, surrounded by inverted terminal repeats (ITR). (**b**) Map of a typical recombinant AAV vector genome, showing replacement of the viral rep and cap genes with a transgene cassette. (**c**) Map of a self-complementary recombinant AAV vector, forming a double-stranded structure due to presence of forward and reverse complement transgene sequences along with one mutated ITR (ITR *).

An adeno-associated virus is a small (~25 nm) parvovirus that contains a single-stranded DNA (ssDNA) with a genome length of 4.7 kilobases, enclosed in a protein capsid [14]. The wild-type AAV genome includes four open reading frames (ORFs). Two of them—rep and cap genes—encode proteins that are essential for genome replication and viral capsid composition, respectively (Figure 1a). Two remaining alternative ORFs are presented by assembly activating protein (AAP) and recently discovered membrane-associated accessory protein (MAAP) encoding genes. The former is involved in capsid assembly, and the function of the latter appears to limit AAV production through competitive exclusion. The entire genome is flanked by two inverted terminal repeats (ITR) [15,16]. Designing a recombinant AAV construct with a gene of interest implies the replacement of rep and cap sequences for transgene expression cassettes, including the transgene sequence as well as regulatory elements. ITRs are obligatory elements of rAAV constructs, since they are important for genome replication and packaging processes during vector production and further expression in target cells. Encoding sequences surrounded with ITRs with a total length of ~5 kilobases, resembling wild-type AAV genome length (Figure 1b), can be encapsidated without a significant loss in packaging efficiency [17].

AAV tropism to the target tissue is explained by a combination of tissue-specific receptors present on the cell plasma membrane. For example, AAV2, AAV3B and AAV6 attach to heparin sulfate proteoglycan. Glycan-conjugated sialic acid is known to interact with AAV1, AAV4, AAV5 and AAV6. AAV9 has a preference for glycans ending in a terminal galactose. Necessary co-receptors have also been reported: the hepatocyte growth factor receptor and human fibroblast growth factor receptor-1 for AAV2; platelet-derived growth factor receptor for AAV5; integrin α5β1, LamR, AAVR and GPR108 for a variety of serotypes [18]. Following internalization by the receptor-mediated endocytosis, the AAV is transported toward the nucleus, where uncoating occurs. After second-strand synthesis, the vector genome is maintained extra-chromosomally (episomally) in the form of unintegrated concatemers. In particular, dsDNA formation is a noticeably rate-limiting process during AAV infection. Maintaining newly synthesized dsDNA stability is also a limiting factor [19]. To overcome these restrictions, self-complementary AAV particles (scAAV) were developed. The genome of scAAV folds into the dsDNA form due to mutation in one ITR, preventing resolution during the replication process. Thus, resulting viral particles skip the second-strand synthesis step within infected cells, and this significantly reduces the waiting time between target cell infection and transgene expression [20,21]. The presence of a complementary sequence in the transgene expression cassette reduces scAAV capacity approximately twofold, up to 2.4 kb (Figure 1c). The maximal length of the encapsidated sequence was reported to be 3.3 kb [22].

Thus, AAV serotypes have different capsid protein sequences, impeding development of a universal method for titration based on recognition by specific antibodies. Encoding sequences vary from one sample to another, but exhibit common features, such as similar length and obligatory presence of ITRs. The simplicity of PCR methods’ customization for transgenes is one reason for the popularity of these methods in the field. Moreover, targeting the ITR sequence during qPCR allows for the detection of a wide variety of rAAV preparations using the same protocol.

## 3. AAV Production

Recombinant AAV production methods differ between research groups. The basic principle of rAAV production implies three genetic constructs delivered to producer cells. The first construct contains the gene of interest flanked with ITRs. The second bears rep/cap sequences and defines serotypes of assembled rAAV. The third introduces helper genes, necessary for AAV genome replication. Depending on the particular production platform, helper factors may be derived from plasmids, helper viruses or stable transformed cell lines. Role of helper virus can be efficiently carried out by adenovirus, introducing E1A, E1B, E2A and E4ORF6 genes, and VA RNA or herpes simplex virus type 1 (HSV-1), providing UL5, UL8, UL52 and UL29 factors, essential for AAV production. Several other viruses, such as members of herpesvirus family, papillomaviruses, as well as baculovirus and human bocavirus 1 are also known to have helper function, wherein their efficacy may be reduced [23]. Despite the ability to generate functional high-yield AAV batches using helper virus-transduced producer cells, safety concerns regarding incomplete helper virus elimination on downstream steps may sometimes compel to choose helper virus-free methods of rAAV production. Therefore a number of expression systems for the production of rAAVs suitable for clinical application were designed and adapted [24]. Today, the predominant platform for rAAV production is transient transfection of human embryonic kidney 293 (HEK293) cells, since two adenovirus genes, E1A and E1B, are integrated into their genome. Producer cell lines of human origin (HeLa, A549 or HEK293) with stable expression of rep and cap genes are also utilized. In this case, the initiation of rAAV production occurs with the introduction of a helper virus. Production systems based on other mammalian cell lines, such as baby hamster kidney (BHK) cells infected with helper viruses, were also reported. A newer platform utilizing baculovirus infection of Spodoptera frugiperda (Sf9) insect cells has recently gained popularity. Manufacturing approaches that use yeast cells to assemble rAAV also seem promising [25]. Currently, no platform appears to be preferred to any other. All of them have their own advantages and limitations, both of which are critically described by Dobrowsky and colleagues [26].

The platform used for rAAV sample production should be taken into account during the development of quantitation and quantification methods, as rAAV characteristics and contaminating agents can differ significantly. For example, the Sf9 insect cell production platform is known to generate a higher percentage of empty and dysfunctional rAAV particles in comparison with other methods. Moreover, Sf9 cells propagate viral particles with different capsid protein modifications and genome methylations than mammalian producer cell lines [27]. On the other hand, a disadvantage of human cell lines is the possibility of cross-contamination with specific human viruses such as HIV or hepatitis, which necessitates the need to set additional QCs. Viruses used for genetic construct delivery to the producer cell line are another source of possible DNA impurities.

To conclude, AAV production systems introduce another challenge for quantification and QC of viral preparations, necessitating many tests to sufficiently characterize different products in terms of safety and efficiency.

## 4. Methods of AAV Quantitation

Quantitative assays provide information about vector titer as one of the most important QC criteria. Titer estimation in crude viral lysates allows us to assess the assembly efficiency of a newly designed vector construction and follow the changes in this parameter after vector genome or capsid modifications. During purification, total rAAV quantity shows efficiency at a particular stage. Accurate quantification in final rAAV preparation is important for applying the known number of vector particles in further tests. The last parameter is of utmost importance for rAAV therapies or preparations undergoing preclinical and clinical trials, since the applied vector dose will determine therapeutic efficacy and product safety [28]. To date, a strongly standardized and generally accepted rAAV titration assay does not exist. Depending on virus particle feature, which is the object of interest for each assay, research methods can be divided into several groups. Western blot, dot blot and enzyme-linked immunosorbent assays (ELISAs) rely on the specific recognition of AAV capsid surface or separate capsid proteins. Multiple cell-based functional tests are used to quantify infectious or transducing units present within rAAV vector preparations [29]. Other procedures are based on AAV genome quantification, such as Southern blotting, intercalating dyes usage or PCR-based methods (qPCR and ddPCR). Values obtained by different assays cannot be compared directly, which often complicates comparisons of results from different research groups and dosage selections. For example, assaying rAAV products with antibodies to whole capsids shows the total number of particles in the analyzed sample; however, these methods cannot distinguish between vectors fully packaged with functional genomes and empty or truncated particles. Tests with intercalating dyes applied directly to preparations are fast and easy to perform, but quantify all nucleic acids presented in a sample, even incorrectly packaged sequences; thus, these approaches are not accurate enough for the main titration method.

The encapsidated AAV genome is a key component of the vector that mediates the transfer of the transgene and, therefore, the functional effect. Titration of vector genomes by quantitative real-time PCR (qPCR) became a widely accepted method for rAAV quantification and clinical dose determination [30,31,32]. Although initial studies reported low levels of qPCR variability [33], obtaining rAAV titers that differed both within a single laboratory and among different laboratories by more than tenfold was demonstrated in subsequent studies [34]. Moreover, applying protocols targeting different sequences may also introduce an inaccuracy [35]. Another PCR-based method, digital droplet PCR (ddPCR), has recently gained popularity [30]. Compared to qPCR, ddPCR is susceptible to fewer variability factors.

### 4.1. Quantitative Real-Time PCR

Quantitative real-time PCR is a type of PCR that enables the detection and measurement of products generated in each cycle of the amplification process. Detection is based on a fluorescence signal that is emitted by DNA-intercalating dyes or fluorescently labeled target-specific probes. The number of cycles when fluorescence intensity crosses the threshold is proportional to the amount of target template added to the reaction [36]. To calculate the exact copy number of viral genomes, one should build a calibration curve with dilutions of samples bearing the same target region. The initial target copy number in calibration standard sample should be defined using another method beforehand.

Measuring AAV titer by means of qPCR was first reported in the late 1990s [37]. Since then, qPCR has rapidly become the standard method of AAV particles’ quantification in purified preparations and raw materials [33,38,39]. Relatively low intra- and interlaboratory variability, sensitivity, specificity, wide range of quantification and simplicity of its performance facilitated its spread; therefore, this method became more convenient for titration AAV vectors than other popular procedures, such as dot blot, ELISA and cell-based assays. However, the selection of primers providing high amplification efficiency and the development of sample preparation protocols for the accurate replicability of the method remain a challenge.

#### 4.1.1. Primer Selection

First protocols of qPCR-based AAV titering assays were designed to target specific sequences of recombinant AAV genomes. CMV promoter-specific primers were reported by Rohr and colleagues [38]. The transgene’s sequence such as eGFP was targeted by Mayginnes and colleagues [39]. These elements were frequently used in AAV constructs at early stages of AAV production development. However, designing therapeutic vectors or sequences for specific laboratory applications necessitates the replacement of the transgene, as well as expression cassette regulatory elements, to reach desired expression levels in target tissues. This requires primers to adapt each time the vector genome changes, which complicates work with a number of constructs, and, furthermore, hinders the comparison of titers between different AAV samples. Thus, targeting transgene sequences appears to be suboptimal at the stage of vector development. At the same time, primers and probes designed for regulatory elements, such as promoters, introns, 5′ and 3′-untranslated regions, as well as PolyA signal sequences, remain useful for titer comparisons of rAAV samples of similar design (Table 1).

A versatile quantitative PCR protocol, targeting conservative sequences in the rAAV genome, could solve the problem of standardization. The only fairly conserved sequences of viral origin in rAAVs are ITRs. Together with this fact, pseudotyping, a major strategy of recombinant AAV genome designing, makes ITR an ideal candidate as a universal qPCR target. AAV2 was the first serotype to be converted to a vector and has since become the most widely studied serotype, especially in preclinical and clinical trials, before other serotypes became available [40,41,42]. Cross-packaging of the AAV2-derived genome into capsids of another serotype was described with the generation of pseudotyped viruses [43]. In 2012, Aurnhammer and colleagues put this principle on the basis of the developed universal protocol for AAV titration [44]. ITRs of AAV serotype 2 were chosen as a target independent of the transgene or regulatory elements of the recombinant AAV genome, as well as chosen serotype (Table 1).

**Table 1:** Examples of primers and probes used for rAAV quantitative analysis.

Applicability	Target	Sequences	References
**Universal** All rAAV samples	ITR2	primers: 5′-ggaacccctagtgatggagtt-3′ and 5′-cggcctcagtgagcga-3′; probe: R-5′-cactccctctctgcgcgctcg-3′-Q.	[44,45,46,47]
**Semi-universal** Group of rAAV samples	CMV promoter	primers: 5′-ttcctacttggcagtacatctacg-3′ and 5′-gtcaatggggtggagacttgg-3′; probe: R-5′-tgagtcaaaccgctatccacgccca-3′-Q; and other sequences.	[46,48,49]
	CAG promoter	primers: 5′-ctgaccgcgttaatcccaca-3′ and 5′-acaagccgtgattaaaccaaga-3′.	[35]
	CBA promoter	primers: 5′-ccgcagccattgccttt-3′ and 5′-ccgcacagatttgggacaa-3′; probe: R-5′-atggtaatcgtgcgagagggcgc-3′-Q.	[30]
	TBG promoter	primers: 5′-aaactgccaattccactgctg-3′ and 5′-ccataggcaaaagcaccaaga-3′; probe: R-5′-ttggcccaatagtgagaactttttcctgc-3′-Q.	[48]
	GRK1 promoter	primers: 5′-tctcttaaggtagccccgg-3′ and 5′-atccgattagatcattctgccc-3′; probe: R-5′-cctcacttttcccctgagaaggaca-3′-Q.	[50]
	RBG intron	primers: 5′-tcaggtgcaggctgcctat-3′ and 5′-tttgtgagccagggcattg-3′; probe: R-5′-agaaggtggtggctggtgtgg-3′-Q.	[30]
	CMV enhancer	primers: 5′-gtcaatgggtggagtatttacgg-3′ and 5′-gcattatgcccagtacatgacct-3′; probe: R-5′-caagtgtatcatatgccaagtacgccccc-3′-Q; and other sequences.	[30,51]
	WPRE	primers: 5′-ttggatgctcgcctgggttg-3′ and 5′-aggaaggtccgctggatcga-3′.	[35]
	SV40 polyA	primers: 5′-agcaatagcatcacaaatttcacaa-3′ and 5′-ccagacatgataagatacattgatgagtt-3′; probe: R-5′-agcatttttttcactgcattctagttgtggtttgtc-3′-Q.	[45,46,47,48]
	BGH polyA	primers: 5′-catataaaatgaggaaattgcatcgca-3′ and 5′-tcagaacccatagagcccaccg-3′; and other sequences.	[35,48,50]
	RBG polyA	primers: 5′-gatttttcctcctctcctgactactc-3′ and 5′-gctgcaggtcgagggatct-3′; probe: R-5′-cagtcatagctgtccctcttctctt-3′-Q; and other sequences.	[30,48]
**Individual** Unique rAAV samples	eGFP	primers: 5′-cacccacgtgaccacccttac-3′ and 5′-ggatgttgcagtcctccctg-3′; and other sequences.	[35,47,48,52]
	hrGFP	primers: 5′-gatccgcagcgacatcaacc-3′ and 5′-gtacaccacctcgaagctgg-3′; probe: R-5′-gaggagatgttcgtgtaccgcgtgg-3′-Q.	[46]
	emGFP	primers: 5′-acggcgacgtaaacggccac-3′ and 5′-gcgaagcactgcacgccgta-3′.	[49]
	pU6	primers: 5′-gggaaataggccctcttcctgccc-3′ and 5′-caccacgtgacggagcgtgac-3′.	[49]
	ITR5	primers: 5′-cccccccaaacgagccag-3′ and 5′-acccccttgcttgagag-3′; probe: R-5′- cgagcgaacgcgacaggggggagagtg-3′-Q.	[47]

R—probe reporter, Q—probe quencher, BGH—bovine growth hormone, CAG—cytomegalovirus enhancer/chicken β-actin, CBA—chicken β-actin, CMV—Cytomegalovirus, eGFP—enhanced green fluorescent protein, emGFP—emerald green fluorescent protein, GRK1—rhodopsin kinase, hrGFP—humanized Renilla reniformis green fluorescent protein, ITR—inverted terminal repeat, polyA—polyadenylation signal, pU6—U6 promoter, RBG—rabbit β-globin, SV40—Simian virus 40, TBG—human thyroxine-binding globulin and WPRE—woodchuck hepatitis B virus posttranscriptional regulatory element.

The versatility of ITR-based qPCR appears to be its main advantage over transgene- or regulatory element-specific titration qPCR assays. However, accumulating data regarding the application of this system to a number of AAV vector preparations revealed its susceptibility to inadequate titer estimation. Depending on the research group, titers defined by ITR-targeting qPCR were underestimated [35,53] or overestimated [47,54] when compared to titers obtained by amplification of internal targets in recombinant genome. These inaccuracies were as high as 10-fold when scAAV was subjected to titration [53]. The underestimation of ITR-derived titers alone can be explained by the presence of truncated vector genomes, where ITRs are absent [30]. Another explanation of titer lowering by ITR-targeting PCR can be non-efficient primer annealing due to secondary structures formed within the AAV genome. Noticeably, the effect of inaccuracy is more noticeable the closer the qPCR target sequence is to the hairpin formed by regular ITR of mutated ITR of scAAV [35,53]. To resolve secondary structures of the vector genome, and, consequently, obtain titer values more similar to those from internal regions of the vector genome, endonuclease digestion of ITR hairpin or betaine addition to reaction may be applied [49,53]. A comparison of ITR-derived titers with those obtained by non-PCR-based methods (for instance, dot blot) also indicated invalid values obtained by ITR-qPCR [44,53]. Possible reasons introducing such deviations are discussed below.

Considering the mentioned inadequacy in titration, ITR-based PCR quantification of AAV vectors requires additional controls for undistorted titer estimation. Nevertheless, the versatility of the method makes it attractive for researchers.

#### 4.1.2. Protocol Modifications

Sample pretreatment is an important part of titration protocols. The first indispensable step is DNase I treatment. This prevents titer overestimation by amplifying non-encapsidated DNA, originating from transfection plasmids or disrupted capsids. Such pre-treatment is even more important when intercalating dye, such as Sybr Green, is used for amplified DNA measurement [39]. To monitor the efficiency of DNAse I treatment, controls with a known copy number of the rAAV genome plasmid treated or not treated with DNase I should be added to the experimental plate [55].

Vector pre-treatment improvements may be continued with the addition of a proteinase K digestion step. Such a modified protocol was described, for instance, by Lock and colleagues [48]. Although it may result in increasing variability of calculated titers, enzymatic capsid digestion led to an increase in measured titer up to 3.5-fold [30,48]. The necessity of proteinase K digestion, at least for some AAV serotypes, was shown later when simple temperature denaturation of capsids prior to amplification was considered ineffective for AAV8, in contrast to AAV2 [46]. This option may be even more critical for the most thermostable capsids, such as AAV5 and AAV1 [56,57]. Contradictory to these studies, DNase I followed by proteinase K treatment significantly (approximately tenfold) lowered PCR-generated AAV titer and increased the variability of assay in research performed by another group [50]. Instead of proteinase K digestion, the addition of Tween to the reaction was proposed in recent work [52]. Such protocol modification resulted in drastically decreased variability in eGFP-targeted qPCR results, and moreover showed low interlaboratory assay variability.

Another group of studies aimed to prevent material loss due to adherence to various materials during titration, particularly at low concentrations. Usually, this includes the addition of surfactant Pluronic F-68 to the buffer used for the dilution of analyzed samples and calibration standards. Utilization of Pluronic F-68 conferred a significant increase in titers, and markedly increased vector quantification accuracy [48,58]. Noticeably, observed signal increase is dependent on vector serotype, which may indicate that AAV capsids of different serotypes differentially attach to plastic [58].

As well as an increase in variability, additional pre-treatment steps may lead to an increased risk of cross-contamination of samples [46]. Subjecting control PBS samples free of AAV vectors either to DNase + proteinase treatment or to multistep column purification showed that AAV-specific sequences were detected in more than half of the processed samples, potentially indicating cross-contamination during sample pre-treatment. Moreover, multistep column purification showed a significantly higher amplification background compared with enzymatic treatment alone [46]. This necessitates, among the established no-template control (NTC), indicating cross-contamination during PCR setup, the introduction of an additional blank control subjected to the same processing steps as the target samples [55].

Despite proposed protocol modifications aimed at minimizing the titer variability discussed above, utilizing a standardized titration protocol is not be the only requirement for comparable results. This is evidenced in a large interlaboratory study, performed by AAVRSM (AAV reference sample material) Working Group during wide characterization of AAV reference materials of serotypes 2 and 8 [34,59]. Final preparations of reference materials were independently assayed by 16 participating laboratories in accordance with standardized qPCR protocol, in which the SV40-polyA sequence was targeted. As a result, significant interlaboratory variation in genome titers was observed, with the coefficient of variation (CV) reaching 77.7%; even within a laboratory, CVs as high as 60% were sometimes noted. A possible explanation for this finding was the use of PCR reagents and primers from different sources [34]. Another consideration is related to incorrect estimation of the plasmid standards concentration, which is used for qPCR calibration curve preparation [30,60].

#### 4.1.3. Calibration Curve

**Figure 2:**
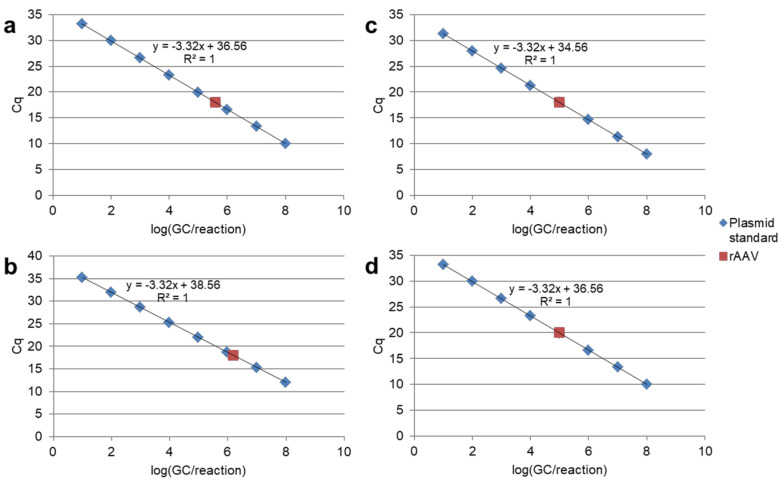
Modeling of incorrect rAAV sample titer estimation due to differences in calibration standard and rAAV sample amplification efficiency. (**a**) Both plasmid calibration standard and rAAV amplify efficiently with real sample titer estimation. (**b**) Plasmid calibration standard is underestimated and rAAV amplifies normally. This leads to sample titer overestimation. (**c**) Plasmid calibration standard overestimated and rAAV amplifies normally. This leads to sample titer underestimation. (**d**) Plasmid calibration standard amplifies normally and rAAV underestimated.

Utilization of different DNA types with known concentrations as a calibration standard can significantly impact rAAV genome quantitation. Standards for rAAV titration can be presented by plasmid DNA, linear DNA fragments, purified viral genomes and viruses themselves. The main attributes of a good standard include the resembling secondary structure and stability to obtain precise and reproducible measurements. Different types of standard sample amplification errors may result in both overestimation and underestimation of rAAV samples. Possible variants are demonstrated on Figure 2. Circular vector plasmids are usually used as calibration standard for rAAV titration [39,53]. At the same time, it has been reported that the amplification efficiency of uncut circular DNA is lower compared to linearized plasmids under identical qPCR conditions, which results in serious overestimations in quantification results [61,62]. The adequacy of circular and linearized plasmid standards was systematically compared in several studies [46,47,49,50]. It was shown that amplification targets on plasmid DNA in close proximity to ITR sequence are more susceptible to inaccurate titration than distal regions, similar to scAAV titration, as discussed above. Furthermore, this effect mostly refers to the circular form of plasmids, and may be unnoticeable for the linearized plasmid standard [49]. Surprisingly, Werling and colleagues (2015) obtained results contradictory to those expected [46]. Their data showed no effect of qPCR amplification suppression on circular plasmids, even for ITR-targeting qPCR. Researchers suggested that protocol details, such as an increased denaturation time, might resolve secondary conformations and therefore increase the amplification efficiency.

A distinct type of linearized vector plasmid, the so-called “free-ITR” calibration standard, deserves special attention [47,49]. Generally, this standard represents an endonuclease-digested linearized plasmid with ITR on both ends, and resembles the rAAV genome; when subjected to qPCR, it shows reduced variability between ITR and transgene or polyA target amplification analysis. Titers generated in protocols containing “free-ITR” calibration were also similar to titers estimated by ELISA. In D’Costa and colleagues (2016), the same work was conducted with ITR of serotype 5 to show that the “free-ITR” approach provides consistent results independently of AAV ITR serotype [47].

Established calibration plasmid standards, as discussed above, share poor structural similarity when analyzing self-complementary vector genomes, and therefore provide inconsistent results [49]. Thus, some authors have proposed the use of distinct standard samples such as the purified scAAV genome. This optimization showed fewer variable results independent of targeted regions of the vector genome, at least when compared to either circular or linear plasmid calibration standards [49].

Finally, denatured rAAV [30] and entire intact viruses [52] were proposed instead of plasmid calibration, aiming to minimize structural differences between analyzing and standard samples. Interestingly, denatured viruses used as standard by Dobnik and co-authors (2019) appeared to be insufficiently stable, and therefore did not solve the high variability problem [30]. On the contrary, intact rAAV is known to be stable during long-term storage, thus providing more consistent results [52]. One limitation that should be noted about vector-based calibration standard is that it provides titers of analyzing samples relative to the previously characterized rAAV standard, and is therefore inappropriate for the initial titration of new rAAV materials. Instead, new vector products should be subjected to titration by existing methods with multiple runs to improve accuracy. Additionally, storage conditions of standard material must be suitable, and stability should be monitored to obtain consistent results.

The importance of choosing calibration standards for accurate dosage measurements of rAAV-based gene therapy is shown in ongoing clinical trials. Substitution of rAAV titration methods during clinical studies of Zolgensma (AveXis) for spinal muscular atrophy treatment led to dose correction. Initial dose 2 × 10^14^ GC, measured by qPCR with supercoiled plasmid as calibration sample, appeared to be equivalent to 1.1 × 10^14^ GC measured by ddPCR [63]. During clinical trials of investigational therapy SRP-9001 (Sarepta Therapeutics) for the treatment of Duchenne muscular dystrophy, analytical method revision was also carried out. The retrospective titration of administered lots, previously measured by qPCR with supercoiled standard by the validated linear standard qPCR method, demonstrated variability, which resulted in a lower dose being received by patients [64].

#### 4.1.4. Reference Material

Unrelated to the chosen type of calibration standard, internal reference rAAV material should be added to each plate when analyzing samples to minimize the interassay variability and to make interlaboratory comparisons viable. This primarily refers to plasmid DNA-based calibration standards due to their lower stability. An additional internal reference sharing maximal structural identity with analyzing AAV vectors may be recommended for the mentioned calibration systems utilizing degraded rAAV or purified rAAV genomes, where DNA is, also, unprotected from degradation. Currently, such references are available for AAV serotypes 2 and 8 from ATTC and other commercial laboratories. However, each laboratory can at least prepare and use their own reference material. For this reason, a highly purified rAAV batch with a known titer should be formulated, aliquoted and properly stored under constant conditions to ensure rAAV stability [47,65].

### 4.2. Droplet Digital PCR

Droplet digital PCR (ddPCR) has recently emerged as a powerful technique for the absolute quantification of AAV. This method utilizes the same chemistry for the detection of amplified target sequences as qPCR, such as DNA-intercalating dyes or fluorescently labeled target-specific probes; thus, it allows for the direct transfer of amplification protocol from qPCR to ddPCR. This approach’s differences in relation to traditional analog PCR lie in sample pre-processing prior to amplification. Rather than amplifying DNA from bulk samples, in droplet digital PCR, individual DNA fragments are partitioned into unique droplets, in which the amplification reactions occur. By partitioning, digital PCR makes it possible to directly count the number of DNA molecules within an initial sample without requiring calibration standards. Another benefit of this method is that, unlike qPCR, the number of AAV vector genomes determined by ddPCR is less affected by the primers and probes used, and it is also less sensitive to inhibitors originating from components in the formulation [66]. This arises from the principle of ddPCR raw data processing, where a fluorescence threshold between clusters of empty and fluorescing droplets can be set independently for each plate well; therefore, producing a sufficient condition for distinguishing positive from negative droplets between clusters is achievable. Its inherent precision, sensitivity and robustness make ddPCR particularly attractive for QC assays [67].

Digital PCR (dPCR) was first introduced in the 1990s [68,69], but only with modern engineering advances has the technique become practical for routine use. In 2014, ddPCR was introduced for AAV titration by Lock and colleagues [48]. These authors reported that ddPCR-derived titers were 2–4-fold higher for ssAAV and 2–8-fold higher for scAAV compared to conventional qPCR, showing lower interassay variation. In contrast, the results of another study indicated ddPCR-derived titer underestimation [30]. These opposing results could be explained by an inaccuracy in plasmid DNA concentration estimation, which then was applied to the qPCR calibration standard curve [30,60]. Later, the results of a study performed by Furuta-Hanawa and co-authors (2019) showed that the ddPCR assay was less susceptible to inaccuracies caused by the secondary structure of the AAV genome. In contrast to qPCR; no significant difference was observed between titers obtained by ITR-ddPCR and polyA-ddPCR assays, and the endonuclease digestion resolving secondary structure of rAAV had no effect on these titers [45]. At the same time, two possible sources of ddPCR-derived result variations should be noted. These include rAAV particle adherence to plastic during the titration procedure or virus aggregation, which can become even worse after the obligatory step of DNase I pre-treatment. In general, it may either disturb the random partitioning of AAV particles in droplets or direct material losses during the set-up, resulting in artificial titer lowering. In a similar vein to how this problem may be solved in qPCR assays, the addition of Pluronic F-68 is recommended in the sample-dilution buffer [30].

In recent years, ddPCR has become the standard technique for industrial use due to its advantages over traditional qPCR techniques, including reduced susceptibility to the presence of impurities and the capacity for absolute quantification without a standard curve [70]. However, ddPCR has its own shortcomings. Unlike qPCR, ddPCR cannot be scaled up from 96 to 384 wells, and each well must be read individually, which limits throughput and prolongs assay time. To date, state-of-the-art approaches allow the simultaneous detection of no more than four fluorescence channels, thereby limiting possible applications of these methods. Nevertheless, further development of ddPCR devices will provide the opportunity to obtain a huge amount of information from one single run. However, the cost of instruments, consumables and reagents are significantly higher than those used in qPCR [71].

### 4.3. Other Methods for Genome Copy Number Measurement

In addition to PCR-based methods, other techniques can be applied for rAAV genome copy number estimation. For example, direct staining of viral samples in agarose gels with fluorescent dyes was proposed, with the aim of determining titers of rAAV without genome amplification [53]. This method implies the separation of denatured vectors on native or denaturing agarose gel alongside a reference virus with a known titer loaded in dilutions. The addition of a reference DNA to each sample, along with subsequent staining with fluorescent dye and densitometry analysis, provides a simple assay with low variation. The sensitivity of the method, as reported in a recent paper, is estimated as 1 × 10^11^ gc/mL [72]. Despite its low specificity, this method can be utilized for the rough assessment of rAAV genomes, with strong secondary structures interfering with the application of more specific assays. The main restriction of titration based on nucleic acid staining is its applicability, limited to highly purified preparations and, also, the error introduced by DNA contaminants of similar size [48].

Another group of methods unites DNA dot/slot blot and Southern blot [73]. Briefly, denatured rAAV is transferred in a series of dilutions to the membrane and hybridized with radioactively labeled probes. These methods also require a standard DNA or vector sample with a known DNA concentration. Although the usage of the DNA probes-based methods appear to be more specific, a narrow detection range limits their application. Considering the particular complexity and sensitivity to operator-induced variability, these methods are therefore unlikely to be adopted as release assays in a commercial production environment [48].

UV spectrophotometry is another simple assay that is applied in rAAV vector genome quantifications [74]. Viral vector absorbance in different wavelengths allows one to define approximate concentrations. Again, the simplicity of performing an assay results in its low specificity. Moreover, this method has high requirements for purity of analyzed samples. Some chemicals remaining after vector purification, as well as protein, DNA or empty capsid impurities, critically affect the adequacy of quantification.

Taken together, non-PCR-based methods of rAAV titration either struggle to provide acceptably low levels of variation or show non-sufficient specificity and are prone to inaccuracy. However, such assays fit perfectly in the initial characterization of newly-generated vectors.

## 5. DNA Impurities

Residual DNA impurities may pose safety concerns for vector preparations. One should pay attention to both free and encapsidated DNA, which is resistant to nucleases during purification and resembles target rAAV particles. Immunogenicity may arise due to induction of innate immunity by free DNA fragments themselves (especially in complex with proteins) or due to the expression of exogenous viral or plasmid DNA, whose products may be recognized by Toll-like receptors [75]. Encapsidated sequences delivered into the target cell along with the intended transgene payload may subsequently integrate, raising genotoxicity risks, or express undesirable sequences inducing immune reactions [76]. Another classification of DNA impurities relies on their source and, therefore, in general, can be divided for process- and product-related impurities. Two abundant sources of process-related DNA impurities are residual nucleic acid constituents of the producer cell line (host cell DNA) and DNA from helper components (plasmids or viruses) used to support vector production. Helper viruses themselves such as adenovirus, herpesvirus or baculovirus may potentially contaminate AAV products when used for vector production and raise infectivity concerns [24]. Truncated vector genomes or restored replication-competent (rc) AAV genomes, as well as other sequences, can be classified as product-related impurities when encapsidated within viral particles. All listed contaminants can be detected by PCR-based methods.

Depending on the production system used, viral oncogenes present within producer cells, such as adenovirus sequences E1 and SV40 large T antigen from HEK293(T) cells or human papillomavirus sequences E6 and E7 from HeLa cells, may remain as impurities in the final vector preparation [77,78]. Importantly, viral oncogene sequences are typically short enough to fit the packaging constraints of AAV capsids. Plasmids sequences, which can remain in viral preparations, usually present bacterial genes such as resistance to antibiotics. This contamination raises concerns both for nucleic acid itself, which can horizontally move to other microbes, leading to the generation of resistant strains and their protein products, as it can induce hypersensitivity reactions in some patients [79]. Contamination with other viruses including helper viruses and pathogenic viruses from producer cell lines also raise concerns. Recently, ddPCR methods were developed for the detection of many viruses, including potential contaminants of rAAV preparations [80]. Another possible contaminant of producer cell lines is mycoplasma [81]. Infection of a cell line with mycoplasma can strongly affect cell morphology and expression profile, bacteria compete for nutrients and therefore reduce the yield of viruses. In addition, some mycoplasmas, including Mycoplasma pneumoniae, are direct risks to the patient’s health if present in the final drug product. PCR analysis for the detection of various mycoplasma species is a convenient method for monitoring contamination both in upstream processes and in the final preparations. Highly sensitive and specific kits for real-time consensus PCR are commercially available [82]. Gene therapy product regulators recommend limiting the amount of free residual DNA to less than 10 ng per dose, and the DNA size to below approximately 200 base pairs [83]. However, this requirement may be difficult to achieve for high doses of vectors. Even a relatively low content percentage of DNA impurity could result in a high absolute number of unwanted sequences [84].

During assembly of rAAV vectors, ITRs function as a key packaging signal. However, the non-vector sequences listed above can also be packaged; this could happen if rAAV vector recombination with non-vector DNA occurs. This process is driven via both homologous and non-homologous recombination. Thus, AAV vector preparations can contain a variety of chimeric sequences that consist of a vector ITR attached to non-vector sequences. A major concern is related to possible recombination between ITR-containing and rep-containing constructs, resulting in replication-competent AAV (rcAAV) particles. They can express rep and cap genes, leading to immunotoxicity, or replicate in the presence of helper viruses [85]. Sequences from vector plasmid backbones can also be packaged by a “reverse packaging” mechanism. To date, adjacent sequences were detected in various AAV preparations, including clinical trial products [86].

The presence of DNA impurities can be quantified by PCR-based systems with primers and probes specific to contaminating sequences (Table 2) [73,87,88,89]. For successfully distinguishing encapsidation from free residual DNA sequences, a DNase digestion step before PCR could be introduced. On the contrary, omitting the DNase digestion step results in total residual DNA, packaged and unpacked, quantification. The current standard assay for residual host cell DNA quantification is based on qPCR or ddPCR analysis targeting highly repetitive genome sequences, such as Alu repeats (Table 2). However, such an analysis cannot provide comprehensive information about undesirable sequences presented in products due to limited coverage of DNA contaminants. Targeting frequently present genomic sequences or fragments of known constructions from introduced plasmids is often profitable; however, the presence of the other sequences will remain unnoticed. Thus, the selection of representative target amplicons is important for assay development. Optimization of PCR workflows and validation against reference materials with known residual DNA profiles could standardize purity tests across laboratories and rAAV products.

The amount of encapsidated DNA impurities in rAAV products is highly dependent on the vector design and manufacturing method. For instance, for scAAV manufacturing by transient transfection of plasmids in human cells, up to 26% of total capsids containing illegitimate DNA was reported [104]; whereas, for ssAAV, these numbers typically do not exceed 1–6% [105,106,107]. For baculovirus expression systems, such amounts were recently estimated at about 2% [108]. Specifically, for plasmid-derived impurities, amount of encapsidated DNA impurities is considered to be influenced by plasmid size, where an oversized (~7 kb) backbone is less likely to be packaged compared its smaller counterpart (~2,5 kb). Thus, the designing of plasmid backbones of larger sizes is recommended [91]. Another strategy may be minicircle technology, where circular DNA expression cassettes do not contain functional or coding prokaryotic sequences, which results in reducing unwanted plasmid sequence contaminations [104].

Another issue concerns the inability to evaluate the presence of a truncated genome. Incomplete forms of vector genome may form during rAAV replication, especially in the case of genomes with strong DNA secondary structures or due to capsid destruction events [109,110]. The design of multiplex PCR-based detection systems targeting different regions of the vector genome could be helpful to attain more information about rAAV genome integrity. Furuta-Hanawa and colleagues (2019) developed a multiplex ITR-polyA ddPCR analysis [45]. When AAV2RSM was analyzed by this protocol, it was noted that, among both ITR- and polyA-positive droplets, there were groups of droplets that were positive for only one target. This observation showed that about 40% of the AAV2RSM particles contained incomplete vector genomes. Moreover, a stability stress-test at 37 °C further increased the presence of such truncated nucleic acids. In contrast, similar single-positive groups originating from SmaI-digested vector plasmid were counted as being less than 2% [45]. Despite the risks mentioned, the negative consequences associated with the detection of encapsulated DNA contaminants have not been confirmed in practice. There is an opinion that the predicted single-stranded nature of misencapsidated DNA impurities renders them unstable and likely to be degraded quickly following unpackaging in the nuclei of transduced cells. This rapid degradation may limit the practical significance of low levels of such impurities as a quality attribute. Thus, further experiments should be performed to confirm or disprove the association of unwanted encapsidated sequences with increased risks of undesirable events. With a clearer understanding, specifications for gene therapy products may be re-considered based on the risk/benefit of a given product [111].

## 6. Complex QC Analysis of rAAV Preparations

PCR methods are widely used for both quantitative and qualitative analysis of rAAV preparations. However, one should critically assess rAAV samples only by their combination with other methods, especially when comparing viral preparations obtained from different production platforms, downstream processing, formulation and storage, taking into account possible impurities and degradation products [112]. QC strategy should be selected based on vector design and application while considering risk assessment [111]. When using primers to ITRs, QC steps should verify ITRs integrity, as they are known to be highly susceptible to recombination events and short deletions. ITR mutations can affect primers’ annealing and amplification efficiency [113], not to mention their impact on production efficiency [114].

One should keep in mind that an equal genome copy number can be measured in samples with different full/empty ratios. As a result, when used in animal experiments, different total capsid contents can influence target cells’ transduction efficiency and immunotoxicity [85]. This can lead to completely different trial consequences. Total capsids content may be as important as the vector genome titer. Quantification of full/empty ratio can be performed using techniques such as ELISA, electron microscopy, analytical ultracentrifugation and high pressure liquid chromatography, as well as many others [30,31,47,115,116,117,118,119,120].

Another example that can affect a sample’s characteristics is measuring the gc number without assessing the percentage of aggregated viral particles. Aggregates can appear in highly concentrated viral samples with low ionic strength and as a result of violations in purification protocol or storage conditions [76]. Aggregation may have deleterious effects on vector transduction efficiency, biodistribution and immunogenicity following in vivo administration, while quantity measured by qPCR remains the same. In contrast, aggregation may influence ddPCR analysis, making partitioning of AAV particles in droplets not random [30]. Methods of viral particle size estimation are mainly based on light scattering detection and are currently being adapted for highly sensitive and reproducing measurements [121]. Differences in functionality of rAAV preparations from different production platforms have been reported. Thus, measuring the same gc number of rAAV samples produced, for example, by transient transfection in an HEK293 cell, and using baculovirus system, will definitely not reflect real infection activity [27]. Differences in many other QC parameters may affect rAAV activity and toxicity, so a combination of methods should be defined accurately based on the source of viral samples and experimental designs.

Some of the QC methods noted above can be set up using commercially available kits and reference samples. Others should be developed for new viral preparations in a sequence- and product-specific manner. During the development of QC methods, researchers should address critical parameters such as linear range, detection limit and susceptibility to specific and non-specific impurities. The majority of methods used for rAAV quantification and QC demonstrate high sensitivity and repeatability in pure viral samples; however, the purification step is not necessary for some applications [122] requiring quantification methods that are highly resistant to cell culture components.

Recent advances in the field of rAAV product approval for medicine applications suggest that current analytical methods are able to sufficiently characterize vector preparations to demonstrate the safety and efficacy of rAAVs in regulatory agencies [11].

## 7. Future Perspectives

A rapidly growing number of clinical trials have highlighted the need to optimize the production of AAV vectors and subsequent processing as cost, among other reasons, led to product discontinuation shortly after the first approval of AAV-based gene therapy [123]. Characterization and quantification are particular challenges in process development and the production of viral vectors. Clinical dosing of rAAV therapeutics is usually based on vector genome titer per mL, thus requiring the availability of accurate QC methods [47]. Despite the number of methods developed for rAAV quantification, PCR-based methods remain more popular. These are widely used and accepted methods for the quantification of AAV vectors due to their simplicity and robustness. Tentative DNA amplification efficiency, which can be significantly impaired by different factors, may be considered as a limitation. These factors include poor design of primers and probes, presence of inhibitors or secondary structures in the target sequences, as noted for self-complementary AAV vectors [53]. To minimize the influence of these factors and interlaboratory differences on obtaining results, selecting, obtaining and detailed descriptions of reference samples are of high importance [124]. Currently, thoroughly characterized reference standards are available for AAV serotypes 2 and 8 [34,59,125] from ATCC (Ref. #VR-1616 and #VR-1816). These can be used both to set up novel methods in the laboratory or to compare titers of home-made reference materials for further routine analysis.

PCR methods are also widely used for the QC of rAAV preparations in terms of process-related impurities that combine host cell products, genetic constructs and contaminating viruses’ quantitation; product-related impurity assessments, which include mispackaged sequences of different origins, are also vital. The key point of PCR methods is the detection of truncated viral genomes, which can be recognized by the majority of PCR-modifications but remain non-functional. For this purpose, qPCR techniques can be accompanied by high throughput sequencing (HTS) applications. Currently, HTS-based methods for the assessment of DNA-related impurities demonstrate higher sensitivity than conventional QC tests based on real-time PCR [105], as well as for the assessment of viral genome integrity [86,126]. For routine analysis, a combination of a few primer pairs should be used to cover different parts of the coding sequence. Indeed, variability in amplification rate due to primer efficiency and secondary structures should be taken into account.

The development and adaptation of ddPCR has revolutionized rAAV quantification and characterization, demonstrating high reproducibility, low sensitivity to impurities and eschewing calibration samples. However, the availability of the necessary equipment and the cost of the procedures preclude many laboratories from deviating from traditional quantitative PCR methods, despite ddPCR and HTS methods representing a considerable development on their predecessors.

## Figures and Tables

**Table 2:** Published primers and probes for contaminating DNA assessment.

DNA Impurity	Target	Sequences	References
Plasmid	KanR	primers: 5′-gggcgcccggttctttttgtc-3′ and 5′-gccagtcccttcccgcttcagtg-3′.	[90]
	AmpR	primers: 5′-cgcgccacatagcagaactt-3′ and 5′-cgccccgaagaacgttt-3′; probe: R-5′-aaaagtgctcatcattg-3′-Q; and other sequences.	[91,92]
	Adenovirus E2A	primers: 5′-ttgctgaaacccaccatttg-3′ and 5′-tcgtggacagcgaggaaga-3′; probe: R-5′-cgccacatcttctct-3′-Q.	[91]
	Adenovirus E4	primers: 5′-tcggcgcactccgtaca-3′ and 5′-cgcgggtctctgtctcaaaa-3′; probe: R-5′-tagggatcgcctacctc-3′-Q.	[91]
	HSV UL23	primers: 5′-tcgatgtgtctgtcctccg-3′ and 5′-atcccatcgccgccctc-3′.	[93]
	HSV UL24	primers: 5′-gccgcgagaacgcgcag-3′ and 5′-cctcgaataccgagcgacc-3′.	[93]
	HSV UL26.5	primers: 5′-catgtccttccacccagac-3′ and 5′-cccatcatctgagagacgaa-3′; probe: R-5′-cagcacacgtggacgttgacac-3′-Q.	[94]
	HSV UL29	primers: 5′-ccgcctatggttaccttgtc-3′ and 5′-ccctcctgtatctggtcgtt-3′; probe: R-5′-agcctcccaggtgcagaaaggt-3′-Q.	[94]
	HSV UL33	primers: 5′- cgaactttacgggacacaatc-3′ and 5′-cgtagtcgggaagacaacct-3′; probe: R-5′-tagacgcgcgctacgtctcg-3′-Q.	[94]
	HSV UL35	primers: 5′-acgcaaacaacacgtttacc-3′ and 5′-tcgaaggttctcgaacgac-3′; probe: R-5′-cggcgcacctattcaccgttt-3′-Q.	[94]
	Ori	primers: 5′-gcgcgtaatctgctgcttg-3′ and 5′-ctacggctacactagaagaacagta-3′; probe: R-5′-cgctctgctgaagccagttaccttcgg-3′-Q.	[45]
	cap8	primers: 5′-tcagccaaggtgggcctaatacaa-3′ and 5′-ttgctgctgcaagttatctgccac-3′; and other sequences.	[90,95]
	rep2	primers: 5′-cggggttttacgagattgtg-3′ and 5′-cgccatttctggtctttgtg-3′.	[95]
	cap2	primers: 5′-ttctcagatgctgcgtaccggaaa-3′ and 5′-tctgccattgaggtggtacttggt-3′.	[96]
Host cell DNA	human Alu	primers: 5′-gaggcgggcggatca-3′ and 5′-cccggctaatttttgtatttttagtag-3′; probe: R-5′-cagcctggccaacatggtgaaacc-3′-Q; and other sequences.	[88,89,97,98,99,100,101]
	Adenovirus E1A	primers: 5′-gggtgaggagtttgtgttagattatg-3′ and 5′-tcctccggtgataatgacaaga-3′; probe: R-5′-agcaccccgggcacggttg-3′-Q.	[102]
	chinese hamster Alu	primers: 5′-agagatggctcgaggttaag-3′ and 5′-tctgcacaccagaagagg-3′; probe: R-5′-agcaccaactgctcttccagagg-3′-Q.	[103]
	Syrian hamster 5S rRNA	primers: 5′-cgcagcagcaggctct-3′ and 5′-accctgcttagcttccgaga-3′; probe: R-5′-ccgccgtcgtctacggccatacc-3′-Q.	[94]
rcAAV	ITR-Rep recombinants	primers: 5′-actccatcactaggggttct-3′ and 5′-gctggggaccttaatcacaa-3′.	[90]

R—probe reporter, Q—probe quencher and HSV—herpes simplex virus.

## Data Availability

Data sharing not applicable.
